# Decreased emergence of HIV-1 drug resistance mutations in a cohort of Ugandan women initiating option B+ for PMTCT

**DOI:** 10.1371/journal.pone.0178297

**Published:** 2017-05-31

**Authors:** Patrycja Machnowska, Andrea Hauser, Karolin Meixenberger, Britta Altmann, Norbert Bannert, Eva Rempis, Alexandra Schnack, Sarah Decker, Vera Braun, Priscilla Busingye, John Rubaihayo, Gundel Harms, Stefanie Theuring

**Affiliations:** 1 Institute of Tropical Medicine and International Health, Charité—Universitätsmedizin Berlin, Berlin, Germany; 2 Division of HIV and Other Retroviruses, Robert Koch-Institute, Berlin, Germany; 3 Holy Family Virika Hospital, Fort Portal, Uganda; 4 Department of Public Health, Mountains of the Moon University, Fort Portal, Uganda; National and Kapodistrian University of Athens, GREECE

## Abstract

**Background:**

Since 2012, WHO guidelines for the prevention of mother-to-child transmission (PMTCT) of HIV-1 in resource-limited settings recommend the initiation of lifelong antiretroviral combination therapy (cART) for all pregnant HIV-1 positive women independent of CD4 count and WHO clinical stage (Option B+). However, long-term outcomes regarding development of drug resistance are lacking until now. Therefore, we analysed the emergence of drug resistance mutations (DRMs) in women initiating Option B+ in Fort Portal, Uganda, at 12 and 18 months postpartum (ppm).

**Methods and findings:**

124 HIV-1 positive pregnant women were enrolled within antenatal care services in Fort Portal, Uganda. Blood samples were collected at the first visit prior starting Option B+ and postpartum at week six, month six, 12 and 18. Viral load was determined by real-time RT-PCR. An RT-PCR covering resistance associated positions in the protease and reverse transcriptase HIV-1 genomic region was performed. PCR-positive samples at 12/18 ppm and respective baseline samples were analysed by next generation sequencing regarding HIV-1 drug resistant variants including low-frequency variants. Furthermore, vertical transmission of HIV-1 was analysed. 49/124 (39.5%) women were included into the DRM analysis. Virological failure, defined as >1000 copies HIV-1 RNA/ml, was observed in three and seven women at 12 and 18 ppm, respectively. Sequences were obtained for three and six of these. In total, DRMs were detected in 3/49 (6.1%) women. Two women displayed dual-class resistance against all recommended first-line regimen drugs. Of 49 mother-infant-pairs no infant was HIV-1 positive at 12 or 18 ppm.

**Conclusion:**

Our findings suggest that the WHO-recommended Option B+ for PMTCT is effective in a cohort of Ugandan HIV-1 positive pregnant women with regard to the low selection rate of DRMs and vertical transmission. Therefore, these results are encouraging for other countries considering the implementation of lifelong cART for all pregnant HIV-1 positive women.

## Introduction

Mother-to-child transmission (MTCT) is the most common way of HIV-1 infection among children under the age of 15[1, 2]. Vertical transmission of the virus can occur during pregnancy, labour and delivery or through breastfeeding. Without any intervention, the risk of transmission ranges between 15% and 45%. Since the end of the last century, the use of antiretroviral (ARV) drugs for the prevention of mother-to-child transmission (PMTCT) has unfolded a potential to reduce the rate of MTCT to <5% [1, 3–5]. However, the frequent emergence of drug resistance mutations (DRMs) linked with temporary single- or combination- ARV regimens has to be viewed as a major threat for future treatment success in mothers and their children by reducing the susceptibility to ARV drugs[6–11]. Even in complex PMTCT regimens with longer intake duration, the level of acquired DRMs remains high as long as they are applied only temporarily, as discontinuing ARV intake after delivery and possible restart during subsequent pregnancies or for later antiretroviral therapy (ART) promotes the emergence of DRMs[12, 13].

Since 2012, the WHO PMTCT guidelines recommend “Option B+”, which stands for the initiation of lifelong combination ART (cART) for all pregnant and breastfeeding women living with HIV-1 regardless of CD4 count or WHO clinical stage[14]. The recommended regimen consists of two nucleoside reverse transcriptase inhibitors (NRTIs) combined with a non-nucleoside reverse transcriptase inhibitor (NNRTI) (first-line regimen), or a protease inhibitor (PI) (second-line regimen). Infants born to HIV-1-infected mothers also receive nevirapine (NVP, an NNRTI) or zidovudine from birth until six weeks of age. While the early start and lifelong intake of cART increases the effectiveness regarding PMTCT by lastingly suppressing viral loads[15], the approach also focuses on improving maternal health and reducing mortality and morbidity of HIV-1 positive women. The continuous intake of cART is reported to decrease or avoid resistance development[16], but suboptimal adherence and loss to follow-up due to individual, structural or community factors still pose a risk for the selection of drug resistance even under Option B+[17, 18]. Besides the need to overcome this huge barrier, surveillance of DRMs is a key factor to optimize treatment outcomes and support national, regional and global decision-making regarding the choice of first-line regimens[6].

Until now, population-based Sanger sequencing has been the gold standard for the screening of HIV-1 genotypic resistance. However, population-based sequencing is not able to detect low-frequency HIV-1 drug resistant variants at levels below 20%[12]. Nevertheless, studies show that also low-frequency HIV-1 drug resistant variants can have an influence on the success of cART[8, 19]. With its high analytical sensitivity, next generation sequencing (NGS) has overcome the limitations of traditional Sanger sequencing and therefore gained increasing importance in the last years[20–22].

Uganda is classified as a HIV-1 high burden country with a prevalence around 7.4% in the general population in 2013[23]. In 2012 Uganda started implementing Option B+ which was scaled-up countrywide since then, increasing the percentage of pregnant women under ART from 86% in 2011 to 94% in 2014[23]. Until now, long-term data on resistance development during intake of Option B+ in a real-life scenario are scarce. In fact, there is only one study from Malawi, reporting DRMs in 8.5% of women at 12 months postpartum (ppm)[24].

The aim of this study was to assess the emergence of HIV-1 DRMs against three major drug classes, NRTIs, NNRTIs and PIs, leading to resistance against the recommended first- and second-line drugs for PMTCT in Fort Portal, Uganda. Using a combination of conventional and real-time RT-PCR assays as well as the NGS platform MiSeq (Illumina) the development and variation in time of DRMs could be assessed.

## Materials and methods

### Ethic statement

Ethical approval was obtained from the Committee of Higher Degrees, Research and Ethics, College of Health Sciences, Makerere University Kampala, by the Ugandan National Council for Science and Technology and by the Ethical Committee of Charité - Universitätsmedizin Berlin. We obtained informed written consent from all participants involved in the study and treated data strictly confidential. Participants could withdraw their study participation at all times without any negative consequences for their treatment in hospital.

### Clinical samples and study design

Between January 2013 and December 2013, women attending antenatal care (ANC) at Virika Hospital and the Regional Referral Hospital in Fort Portal, Uganda, were recruited for a longitudinal prospective cohort study as a part of a larger study on Option B+ outcomes. According to the WHO and Ugandan PMTCT guidelines from 2012, HIV-1 positive pregnant women were enrolled on Option B+, composed of a single-pill based on a NRTI/NNRTI, fixed-dose combination of efavirenz/lamivudine/tenofovir (EFV/3TC/TDF) as first-line regimen; newborns received NVP from birth up to six weeks of life, regardless of infant feeding method[14]. Women were included in the study due to following eligibility criteria: Informed written consent, age ≥18 years, confirmed pregnancy and positive HIV-1 status without prior ART enrolment.

Women were followed-up at subsequent ANC and postpartal care visits until 18 ppm. Plasma samples from women were collected at the first ANC visit before initiating Option B+ (baseline sample), at subsequent ANC visits, at six weeks postpartum (ppw) as well as six, 12 and 18 ppm. From infants, plasma samples were obtained or blood from heel pricks was spotted onto filter papers (Dried Blood Spots, DBS) at the same time intervals. In the present study, which aims to analyse the emergence of DRMs at least 1 year after initiation of Option B+ in ANC only women with a sample at 12 and/or 18 ppm and a respective baseline sample available were included.

### Extraction of viral nucleic acids

Nucleic acids were extracted using the NucliSens easyMAG system (Biomerieux) according to the manufacturer`s instructions. Briefly, plasma samples were centrifuged for 10 min at 4°C with 3000 g to remove cryoprecipitate. Subsequently, 500μl of plasma were transferred into 2 ml of lysis buffer, mixed and incubated at room temperature for 10 min. After adding 100 μl of magnetic silica solution, total nucleic acids were extracted by automated magnetic separation (Generic 2.0.1) and finally eluted in 60 μl of elution buffer. Extracted nucleic acids were aliquoted in portions of 10 μl and immediately stored at -80°C until further use.

For the extraction of total nucleic acids from DBS, 2 blood spots (50 μl blood each) were cut out from the filter paper and transferred to 2 ml of lysis buffer. After shaking for one hour at room temperature with 300 rpm, 1.8 ml of lysated eluate was used for automated nucleic acid extraction performed as described above.

### Amplification of nucleic acids

For amplification of viral RNA three different PCRs were performed; two conventional RT-PCR assays to identify DMRs in the protease (PR) and reverse transcriptase (RT) genomic region of the HIV-1 polymerase (*pol*)-region and a real-time TaqMan RT-PCR to determine the HIV-1 viral load. With the priority of obtaining as many sequences as possible for DRM analysis and due to the limited material available, first conventional RT-PCR assays were performed, followed by HIV-1 viral load measurement.

For conventional RT-PCR two overlapping fragments of 576 bp length (fragment 1) containing the codons 1 to 99 of the PR and codons 1 to 93 of the RT and of 718 bp length (fragment 2) containing the codons 22 to 260 of the RT [25] were amplified using the Qiagen One Step RT PCR kit and an Eppendorf Mastercycler pro. The resulting PR-RT sequence (fragment 1+2, position 2277–3302 on HXB2 genome, Acc. No. K03455.1) covers all resistance-associated mutations of PR and RT. In the following text viral sequences named “PR”, “RT” and “PR-RT” refer to the corresponding PCR-product of “fragment 1, 2 or 1+2”.

Conventional RT-PCR assays were performed on all maternal 12 and 18 ppm samples as well as respective baseline samples. Additional samples from earlier time points (6 ppw or 6 ppm) were subjected to the PCRs if maternal HIV-1 sequences displayed DRMs. Samples resulting in RT-PCR fragments were applied to NGS. Infant 12/18 ppm samples were analysed by conventional RT-PCR. In case of a positive result infants were considered to be HIV-1 infected.

The HIV-1 viral load was determined using a quantitative real-time TaqMan RT-PCR (MX3005P qPCR system from Agilent) with primers located in the long terminal repeat (LTR) of the HIV-1 genome. Primers and probe for the amplification of a 118 base pair (bp) fragment were used as described by Cleland et al., 2001[26]. An external standard curve generated from known concentrations of the laboratory strain HTLVIIIB (Acc. No. K03455.1) was carried out on each plate to enable absolute quantification of HIV-1 genome equivalents in every sample. Viral load was determined for all maternal baseline samples and post partum samples resulting in RT-PCR fragments. In this study, samples with HIV-1 viral loads >1000 copies/ml were defined being a result of “virological failure” (VF).

### Next generation sequencing (NGS)

Amplicons were purified using Agencourt AMPure® XP Beads (Beckman Coulter) and quantified using Quant-iT™ PicoGreen® dsDNA Reagent (Invitrogen). The Nextera XT® kit (Illumina) was used to prepare the NGS libraries. Sequencing was performed on the Illumina MiSeq® employing the 2 x 200 or 2 x 300 bp paired end modus. The NGS data were processed through an in-house bioinformatics pipeline making use of the tools Trimmomatic (version 0.33)[27], FLASH (version 1.2.11)[28], and BWA (version 0.7.10)[29], all wrapped in a Python script. After adapter-clipping, trimming and merging, five iterations of mapping against a reference sequence (HXB2, Acc. No. K03455.1) were performed. Potential insertions and deletions were considered during the mapping cycles. The final output was subject to further Python scripts, which enabled codon-based variant detection and generation of a consensus sequence containing ambiguities according to an adjustable threshold. A Sanger-like threshold of 20% was applied in general. Moreover, the sensitivity and specificity for the detection of low-frequency drug resistant variants was determined using mixtures of recombinant HIV-1 from the wild-type pNL4.3 clone (Acc. No. M19921) and a mutant pNL4.3 derived clone that harbours twelve DRMs within *pol*. Cloning and generation of the recombinant virus has been described previously[6, 30]. Twenty-eight mixtures ranging from 1% to 50% mutant in wild type were prepared in viral loads ranging from 10^3^ to 10^6^ copies/ml. In mixtures with viral loads <10^5^ copies/ml, the sensitivity of detection varied and the noise was higher than in mixtures with viral loads ≥10^5^ copies/ml. Consequently, NGS thresholds for low-frequency variants of 3% and 10% for samples with low and high viral load, respectively, were determined.

### Detection of viral drug resistance mutations and determination of HIV-1 subtype

Only samples with successfully generated sequence were included in the final DRM-analysis. DRMs were determined according to the Stanford University’s HIV-1 drug resistance database[31] and by the International Antiviral Society USA (IAS-USA)[32].

The prevalence of pre-existing transmitted drug resistance mutations (TDRMs) in baseline samples was assessed using the WHO surveillance drug resistance mutations list (SDRM)[33]. Therefore, resistance mutations in the PR and RT were considered.

In contrast, the prevalence of DRMs selected by the implemented NRTI/NNRTI regimen was only analysed in the viral RT sequence of post partum samples, as no selection of DRMs in PR was expected. Based on sequence availability, comparison of viral RT sequences from baseline samples to respective sequences from follow-up (FUP) samples after delivery was performed to identify emerging DRMs acquired during the PMTCT regimen. However, the extent of DRM-selection in FUP samples without the respective baseline sequence could be assessed by monitoring their proportion compared to the respective 6 ppw and 6 ppm samples. Drug resistant low-frequency variants were defined as DRMs detected by NGS below the Sanger-like cut-off of 20%.

HIV-1 subtypes were determined on basis of the Sanger-like NGS sequences with an ambiguity threshold of 20%. HIV-1 subtypes were determined using the REGA HIV-1 subtyping tool v3.0[34].

## Results

### Study population

124 women attending ANC between January and December 2013 were tested HIV-1 positive. All women were drug naïve at enrolment and received the recommended first-line regimen consisting of EFV/3TC/TDF. Socio-demographic and clinical baseline data, as well as information on the antenatal adherence of the study group were published by Schnack et al., 2016[35].

**Fig 1:**
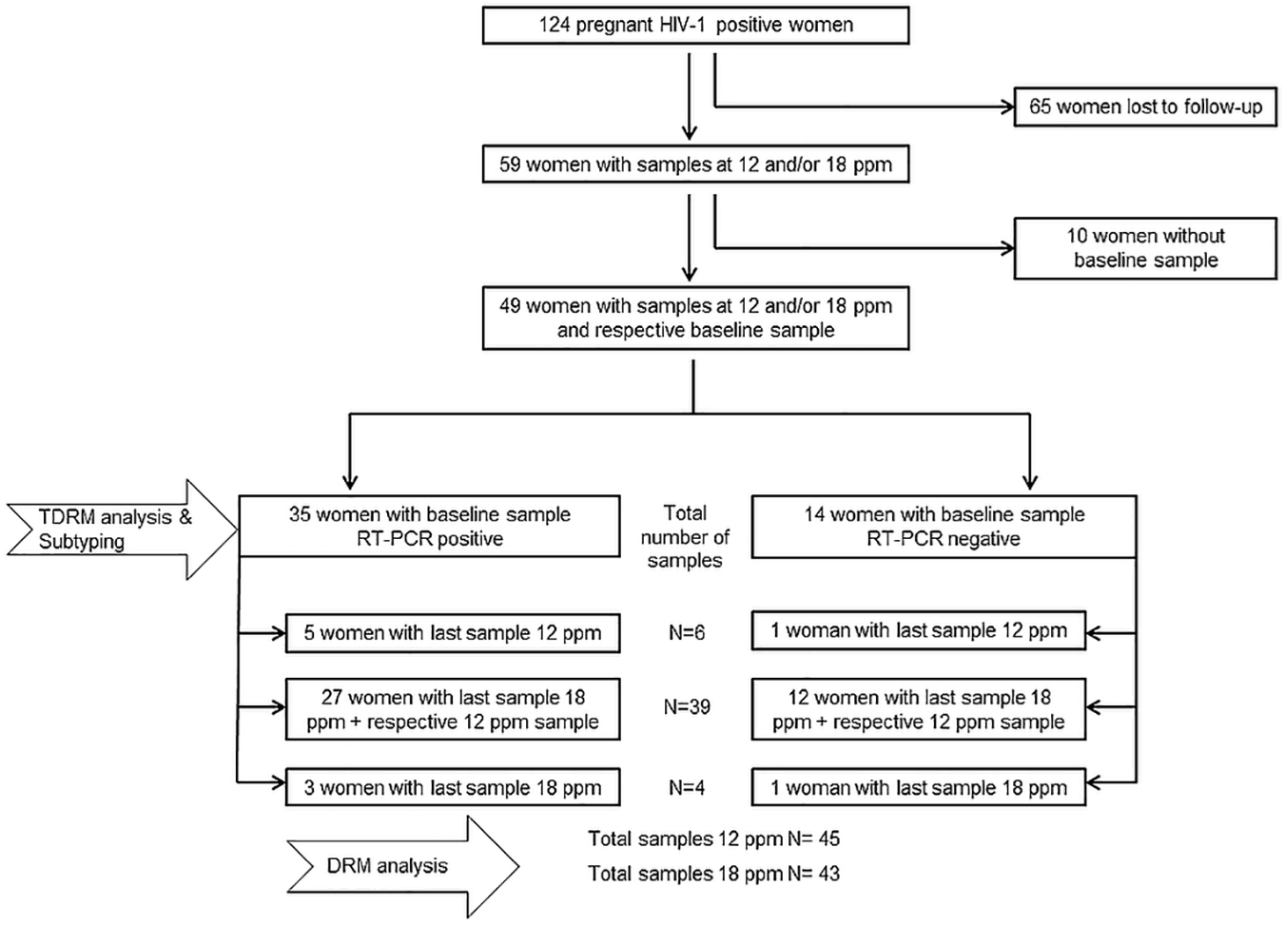
Sample availability.

Only 59/124 (47.6%) women completed the study-period at 18 or at least 12 ppm. However, ten of them were excluded due to missing baseline samples. These 49 women finally constituted to the present study cohort for genotypic resistance testing (Fig 1). Both, the 12 and 18 ppm sample were available for 39 women, while either the 12 or 18 ppm sample was available for six and four women, respectively, resulting in overall 49 women with 45 samples from 12 ppm and 43 samples from 18 ppm. Baseline, 6 ppw and 6 ppm samples were available for 49/49, 43/49 and 44/49 women, respectively (6 ppw and 6 ppm not shown in Fig 1).

Analysing the last available sample of involved infants, in 43 infants at 18 ppm and six infants at 12 ppm, no HIV-1 infection was detected.

### Genotypic resistance testing and virological failure

Assessment of TDRMs was carried out for 35/49 RT-PCR positive baseline samples, resulting in 27 PR-RT sequences, six PR sequences, and two RT sequences (Table 1).

**Table 1:** Outcome sample analysis.

	N (%)	No. PCR	Viral load tested for	Succesful NGS for
		positive of	No. of samples and	No. of samples
		No. analysed	median viral load (IQR)	analysed and
			in copies/ml	sequence results
**No. of women included in**	49			
**DRM analysis**				
**Baseline samples for No. of women**	49 (100)	35/49	42/49	35/35
			4.9x10^3^	27 PR-RT
			(1.2x10^4^-1.1x10^3^)	6 PR
				2 RT
No. of samples taken at time point				
**6 ppw**	43 (87.8)	2/3	2/2	2/2
			4.1x10^3^	2 PR-RT
			(5.1x10^3^-3.2x10^3^)	
**6 ppm**	44 (89.8)	3/3	3/3	3/3
			1.5x10^4^	3 PR-RT
			(1.8x10^4^-9.3x10^3^)	
**12 ppm**	45 (91.8)	5/45	5/5	5/5
			1.1x10^4^	5 PR-RT
			(1.3x10^4^-0)	
**18 ppm**	43 (87.8)	7/43	7/7	6/7
			1.1x10^4^	5 PR-RT
			(5.2x10^4^-4.3x10^3^)	1 RT

No.: number.

Furthermore, PR-RT sequences could be obtained for 2/3 samples at 6 ppw, 3/3 samples at 6 ppm, 5/5 samples at 12 ppm and 5/7 samples at 18 ppm. One 18 ppm sequence covered the RT fragment only and one sequence generated by NGS failed the inclusion criteria due to a low number of reads. In total 52 samples were analysed by NGS. The mean read length ranged from minimal 137 to maximal 219 bp. After pre-processing, ~18.000 to ~537.000 valid reads led to mean coverages of ~1.100 to ~126.000.

Viral loads could be detected in 42/49 (85.7%) baseline samples (median 4.9x10^3^; IQR 1.2x10^4^-1.1x10^3^), in 3/5 12 ppm samples and 7/7 18 ppm samples. For all three samples with VF, defined as viral loads >1000 copies/ml, at 12 ppm the respective 18 ppm sample likewise displayed VF. In total, 3/45 and 7/43 women at 12 and 18 ppm (corresponding to 7/49 women at 12/18 ppm) experienced VF corresponding to 93.3% and 85.7% of women at 12 and 18 ppm reaching viral suppression.

### Drug resistance mutations at baseline and subtyping

According to the sequence availability of baseline samples TDRMs in the PR and RT of HIV-1 could be analysed for 33 and 29 of 35 women, respectively (Table 1). In none of the 29 RT-sequences NRTI- or NNRTI-selected mutations according to the WHO-SDRM list[33] could be detected. Likewise, no PI-selected TDRMs were found in the 33 PR-sequences. However, considering the IAS-USA mutation list[32] the Q58E mutation in the PR was detected in one sample at baseline. Furthermore, a large number of minor PI resistance mutations were found in the baseline samples[32]. The most prevalent minor PI resistance mutation was M36I (31/33), followed by H69K (24/33) and L89M (20/33). Further detected minor PI resistance mutations were L10I and L10V (each 1/33), G16E (4/33), K20I and K20R (4/33 and 10/33, respectively), 33 (1/33), D60E (4/33), I62V (5/33), L63P (11/33), I64V and I64M (10/33and 1/33, respectively), V82I (3/33) and I93L (1/33).

HIV-1 subtypes were determined from PR-RT baseline sequences of 35 women. Subtype A1 was predominant in the study cohort (19/35; 54.3%), followed by subtype D (7/35; 20.0%). 14.3% (5/35) of women were infected with recombinant HIV-1 strains, while 8.6% (3/35) and 2.9% (1/35), were infected with subtype G and C HIV-1, respectively.

### Drug resistance mutations at 12 and 18 ppm

HIV-1 RT genomic region could be analysed for 5/5 12 ppm and 6/7 18 ppm samples, corresponding to seven women (Table 1). DRMs were detected in 3/5 12 ppm and 3/6 18 ppm HIV-1 samples (Table 2), corresponding to three women. Hence, HIV-1 DRMs were detected in 3/49 (6.1%) women at 12 and/or 18 ppm.

**Table 2:** Outcome of detection of DRMs in three women.

Sample		Baseline	6 ppw	6 ppm	12 ppm	18 ppm
ID						
**1**	**VL**	1.2x10^4^	6.0x10^3^	1.5x10^4^	1.3x10^4^	4.8x10^4^
	**(copies/ml)**					
	**RT-PCR**	pos	pos	pos	pos	pos
	**DRMs (%)**	wt	wt	*K103N (16.8)	**K103N (6.32)	K103N (82.2)
**2**	**VL**	5.5x10^4^	-	3.5x10^3^	1.1x10^4^	5.8x10^3^
	**(copies/ml)**					
	**RT-PCR**	pos	neg	pos	pos	pos
	**DRMs (%)**	wt	ns	K70E (99.6)	K70E (99.7)	K70E (99.7)
				M184V (99.4)	M184V (99.5)	M184V (96.8)
				K103N (99.2)	K103N (99.1)	K103N (99.4)
				E138Q (99.3)	E138Q (99.5)	V108I (29.6)
					*P225H (13.6)	E138Q (99.6)
						P225H (76.2)
**3**	**VL**	2.0x10^4^	2.2x10^3^	2.1x10^4^	1.7x10^4^	1.3x10^5^
	**(copies/ml)**					
	**RT-PCR**	neg	pos	pos	pos	pos
	**DRMs (%)**	ns	M184V (85.4)	M184V (94.7)	M184V (97.2)	M41L (41.7)
			K103N (84.2)	T215Y (21.5)	**L210W (4.3)	L74V (35.0)
			V179T (32.2)	K103N (89.4)	T215Y (90.4)	M184V (99.4)
				*V179T (13.0)	K103N (99.4)	L210W (71.2)
						T215Y (99.5)
						*A98G (4.4)
						L100I (93.8)
						K103N (98.9)
						*V108I (6.0)
						*V179T (3.7)
						P225H (20.2)

VL: viral load; wt: wild type; nd: not detected; ns: no sequence from sample; -: exclusion according to sample inclusion criterion

* Low-frequency drug resistant variant

** Low-frequency drug resistant variant below the NGS cut-off, defined dependent on viral load.

In one woman (Sample ID 1) HIV-1 resistance against EFV and NVP caused by the K103N mutation in the RT was selected. This woman had reported full adherence until 12 ppm and stated at 18 ppm not having taken pills for >seven days. The two other women (Sample ID 2 and 3) showed dual-class drug resistance against NRTIs and NNRTIs, including all drugs recommended for first-line ART. In both women, mutations K103N, P225H and M184V were detected, although they reported full adherence until 18 ppm. Outcome of the DRM analysis for the remaining 46 women is listed in S1 Table.

Low-frequency drug resistant viral variants were found in only one of the three women at 18 ppm (Sample ID 3: V108I 6.0% and A98G 4.4%). At 12 ppm, low-frequency drug resistant variants were detected in another woman (Sample ID 2: P225H 13.6%). Furthermore, low-frequency drug resistant variants–but below the defined cut-off dependent on the viral load of the sample—were also found in two women at 12 ppm (Sample ID 1: K103N 6.3% and sample ID 3: L210W 4.3%). Nevertheless, all low-frequency drug resistant variants found at 12 ppm, independent if below or above the defined NGS cut-off, were detected in the respective 18 ppm sample selected as main viral variant (Table 2). All women with DRMs at 12/18 ppm were of subtype D.

## Discussion

Our study is among the first to provide data on the selection of DRMs until at least 1 year after initiation of Option B+ for PMTCT.

According to WHO recommendations the success of HIV-1 drug treatment is defined by a viral load suppression in >85% of individuals after 12 months of ART [36]. This criterion was met in our study with 93.3% (42/45) of women at 12 and 85.7% (36/43) at 18 ppm reaching viral suppression. First results of women enrolled in Option B+ in Malawi (recommended first-line regimen: EFV/3TC/TDF) report viral suppression in 91.5% of women at 12 ppm, which is comparable to our results[24]. Data from other countries implementing Option B+ are not available until now. However, in another study from Malawi, where pregnant women initiating cART between 2008 and 2011 were followed up until 24 ppm, viral suppression was achieved in 87.1% of women at 12 and 89.9% at 18 ppm, respectively[37]. In a cohort of pregnant women in Tanzania, women initiating cART between 2004 and 2006 had a viral suppression rate of only 47% at 12 ppm[38]. However, in both studies, women initiated cART for their own health due to a low CD4 cell count at baseline. Furthermore, in both settings an NVP-based drug regimen was the first-line recommendation at the time of study. Therefore, these data are not directly comparable to the data from our study, where drug naïve women initiated cART independent of CD4 cell count. The high virological suppression rates in our study as in Malawi support the latest WHO guidelines moving away from different “options” for PMTCT to the early, lifelong cART initiation for all pregnant HIV-1 positive women regardless of clinical or CD4 cell count or stage of disease[39]. Nevertheless, a known problem assessing the potential success of PMTCT interventions in low-income settings is the high number of women lost to follow-up[40]. Taking into account the loss to follow-up rate of 52.4% in this study, the true viral suppression rate is probably much lower and fails to meet the criteria for successful HIV-1 drug treatment. Furthermore, using the reversed approach of testing in our study–conventional RT-PCR followed by viral load measurement–we might have missed women with detectable viral load (due to the sensitivity of the conventional RT-PCR of ≥1000 copies/ml). However, the application of both assays (RT-PCR and viral load measurement) to all baseline samples revealed only 7/49 RT-PCR negative samples with viral load (which was below 1000 copies/ml for all seven cases) indicating that all women experiencing VF were identified by our testing scheme.

It has been shown that stopping and restarting an NNRTI-based regimen poses a risk for emergence of DRMs and may influence suppression rates when restarting a similar regimen[13, 41]. In our study, DRMs were detected in 6.1% (3/49) of women at 12 and/or 18 ppm. The absence of DRMs in the sequence of baseline samples of two women (Sample ID 1+2) enabled to identify the DRMs at 12/18 ppm as newly acquired (Table 2). For the third woman (Sample ID 3) the corresponding baseline viral sequence was missing due to a negative result in the two conventional RT-PCR assays. However, increasing levels of DRMs from 6 ppw up to highest levels at 18 ppm points to an ongoing selection of DRMs. Among women displaying VF almost half of them (42.9%; 3/7) selected for HIV-1 DRMs. Two of them harboured multiple DRMs, resulting in dual-class resistance against NRTIs and NNRTIs, including those recommended as first-line therapy for Option B+ (EFV/3TC/TDF). Although all three women reported full or almost full adherence, the selection of drug resistance mutations as well as the nearly constant detection of viral loads above 1000 copies/ml in the follow-up samples indicate suboptimal drug concentrations. However, besides drug adherence, other factors like pharmacogenomic issues or drug interactions with additional therapeutic agents against co-morbidities might contribute to changes in drug levels or drug half life, resulting in the development of DRMs[42]. Furthermore Kyeyune et al., observed a higher frequency of treatment failure and drug resistance in subtype D versus subtype A infected individuals[43]. All three women experiencing treatment failure in this study were of subtype D indicating a possible influence of the subtype, maybe in combination with other adverse factors, on treatment success. However, to confirm this hypothesis a closer examination of more subtype D infected individuals with and without treatment failure would be required. The NNRTI-associated mutation K103N was observed in all these three women remarkably present as the main viral variant. In Malawi, where low-frequency HIV-1 drug resistant variants were not analysed, DRMs were present in 8.5% of women at 12 ppm, corresponding to a comparable percentage of 40% for women with detectable viral load. In line with our results, they also detected the K103N in all women with DRMs. In the above mentioned studies from Tanzania and Malawi with women initiating life-long cART due to low CD4 counts, DRMs were observed in 34% and 6% of women at 12 ppm, respectively[37, 38]. However, in the Malawian cohort only 15/27 samples with detectable HIV-1 RNA were sequenced, which possibly leads to underestimation of the DRM. Data from the pre-Option B+ era report DRMs (including low-frequency variants) in 40% of infected Tanzanian women receiving a drug regimen resembling Option A between delivery and 16 weeks postpartum[6]. Compared to these findings, the rate of 6.1% of women with DRMs at 12 and/or 18 ppm in this study and 8.5% at 12 ppm in Malawi clearly show the benefit over earlier PMTCT recommendations. Nevertheless, the high number of women lost to follow-up in this study (65/124; 52.4% of women) has to be considered. Women lost to follow-up probably also may have been less adherent to the regimen thereby impeding treatment success. Therefore, the true percentage of DRMs is difficulty to assess and is probably underestimated in this study analysing the more adherent women.

Low frequency variants of NNRTI-associated DRMs were detected in two of three women at 12 and 18 ppm. However, their contribution to VF cannot be assessed, as they were found among multiple DRMs. Several studies indicated that low-frequency drug resistant variants are associated with VF[19]. In a recent systematic review of literature it has been shown that individuals with low-frequency drug resistant variants and <95% medication adherence had a 5.1 times higher risk of VF compared to individuals with low-frequency drug resistant variants and a medication adherence of >95%[44]. Kyeyune et al., reported the presence of DRMs in low frequency only for 65% of Ugandan patients failing cART [45]. However, in other studies no association between VF and the presence of low-frequency drug resistant variants was observed[46, 47].

No TDRMs according to the WHO SDRM list were detected in women at baseline in our study cohort. However, based on the algorithm of the IAS-USA, a number of minor PI-resistance mutations were detected at baseline. These mutations are common non-B subtype polymorphisms, some of which have been associated with early treatment failure and higher number of acquired major PI-associated mutations at time of treatment failure under a PI containing regimen[48–50]. This should be kept in mind when switching to a PI containing second-line regimen, as treatment success might me impaired. In total, the prevalence rate of TDRMs in this study was 0%, although highly sensitive NGS was performed. Other studies from Uganda report varying rates of TDRMs between 1.5% and 19% with respect to different populations, regions and surveillance strategies[51, 52]. In pregnant ART-naïve women, two studies between 2006 and 2007 observed TDRM rates ranging from 0% to 5%, respectively[53, 54]. These data are in line with the rate found in our study cohort.

The small sample size is a major limitation of our study as only 49 women with a respective baseline sample could be followed-up until 12/18 ppm. However, limited samples sizes in the context of HIV-1 positive cohort FU are a common problem in these settings, while at the same time, our data are among the first to describe the development of DRMs in HIV-1 positive pregnant women starting Option B+ in a real-life scenario. For a growing number of countries, now implementing the lifelong intake of cART for PMTCT, these data are encouraging with regards to DRMs linked with the initiation of an early lifelong treatment for all HIV-positive pregnant women. For the future, data from large-scale, multicentre studies assessing the emergence of DRMs during Option B+ will be required to confirm the findings of this research.

## Supporting information

S1 Table Outcome of detection of DRMs in 56 women. VL: viral load; wt: wild type; nd: not detected; ns: no sequence from sample; -: absence of sample, or to exclusion according to sample inclusion criterion.
(DOCX)
