# Supplementary material for: Decreased emergence of HIV-1 drug resistance mutations in a cohort of Ugandan women initiating option B+ for PMTCT
Source: PLoS One. 2017 May 31;12(5):e0178297. doi: 10.1371/journal.pone.0178297 (PMC5451067; doi:10.1371/journal.pone.0178297)
Supplement: S1 Table — VL: viral load; wt: wild type; nd: not detected; ns: no sequence from sample; -: absence of sample, or to exclusion according to sample inclusion criterion. (DOCX) [file pone.0178297.s001.docx]

| **Sample ID** |  | **Baseline** | **6 ppw** | **6 ppm** | **12 ppm** | **18 ppm** |
| --- | --- | --- | --- | --- | --- | --- |
| **4** | **VL (copies/ml)** | 2.8x10^3^ | - | - | - | 1.1x10^4^ |
|  | **RT-PCR** | pos | - | - | - | pos |
|  | **DRMs (%)** | wt | - | - | - | wt |
| **5** | **VL (copies/ml)** | 9.1x10^3^ | - | - | - | - |
|  | **RT-PCR** | pos | - | - | neg | neg |
|  | **DRMs (%)** | wt | - | - | ns | ns |
| **6** | **VL (copies/ml)** | 8.0x10^3^ | - | - | - | - |
|  | **RT-PCR** | pos | - | - | neg | neg |
|  | **DRMs (%)** | wt  41m | - | - | ns | ns |
| **7** | **VL (copies/ml)** | 8.0x10^3^ | - | - | - | - |
|  | **RT-PCR** | pos | - | - | neg | neg |
|  | **DRMs (%)** | wt | - | - | ns | ns |
| **8** | **VL (copies/ml)** | 4.1x10^3^ | - | - | - | - |
|  | **RT-PCR** | pos | - | - | neg | neg |
|  | **DRMs (%)** | wt | - | - | ns | ns |
| **9** | **VL (copies/ml)** | 7.1x10^3^ | - | - | nd | 2.7x10^3^ |
|  | **RT-PCR** | pos | - | - | pos | pos |
|  | **DRMs (%)** | wt | - | - | ns | wt |
| **10** | **VL (copies/ml)** | 5.7x10^3^ | - | - | - | - |
|  | **RT-PCR** | pos | - | - | neg | neg |
|  | **DRMs (%)** | wt | - | - | ns | ns |
| **11** | **VL (copies/ml)** | 1.3x10^3^ | - | - | - | - |
|  | **RT-PCR** | pos | - | - | neg | neg |
|  | **DRMs (%)** | wt | - | - | ns | ns |
| **12** | **VL (copies/ml)** | 2.0x10^5^ | - | - | - | - |
|  | **RT-PCR** | pos | - | - | neg | neg |
|  | **DRMs (%)** | wt | - | - | ns | ns |
| **13** | **VL (copies/ml)** | 4.3x10^1^ | - | - | - | - |
|  | **RT-PCR** | pos | - | - | neg | neg |
|  | **DRMs (%)** | wt | - | - | ns | ns |
| **14** | **VL (copies/ml)** | 1.04x10^3^ | - | - | - | - |
|  | **RT-PCR** | pos | - | - | neg | neg |
|  | **DRMs (%)** | wt | - | - | ns | ns |
| **15** | **VL (copies/ml)** | 3.9x10^5^ | - | - | - | - |
|  | **RT-PCR** | pos | - | - | neg | neg |
|  | **DRMs (%)** | wt | - | - | ns | ns |
| **16** | **VL (copies/ml)** | 5.1x10^2^ | - | - | - | - |
|  | **RT-PCR** | pos | - | - | neg | neg |
|  | **DRMs (%)** | wt | - | - | ns | ns |
| **17** | **VL (copies/ml)** | 2.8x10^3^ | - | - | - | - |
|  | **RT-PCR** | pos | - | - | neg | neg |
|  | **DRMs (%)** | wt | - | - | ns | ns |
| **18** | **VL (copies/ml)** | 1.7x10^3^ | - | - | - | - |
|  | **RT-PCR** | pos | - | - | neg | neg |
|  | **DRMs (%)** | wt | - | - | ns | ns |
| **19** | **VL (copies/ml)** | 1.1x10^5^ | - | - | - | - |
|  | **RT-PCR** | pos | - | - | neg | neg |
|  | **DRMs (%)** | wt | - | - | ns | ns |
| **20** | **VL (copies/ml)** | 1.0x10^4^ | - | - | - | - |
|  | **RT-PCR** | pos | - | - | neg | neg |
|  | **DRMs (%)** | wt | - | - | ns | ns |
| **21** | **VL (copies/ml)** | 4.0x10^4^ | - | - | - | - |
|  | **RT-PCR** | pos | - | - | neg | neg |
|  | **DRMs (%)** | wt | - | - | ns | ns |
| **22** | **VL (copies/ml)** | 3.2x10^3^ | - | - | - | - |
|  | **RT-PCR** | pos | - | - | neg | neg |
|  | **DRMs (%)** | wt | - | - | ns | ns |
| **23** | **VL (copies/ml)** | 7.0x10^3^ | - | - | - | - |
|  | **RT-PCR** | pos | - | - | neg | neg |
|  | **DRMs (%)** | wt | - | - | ns | ns |
| **24** | **VL (copies/ml)** | 6.6x10^0^ | - | - | - | - |
|  | **RT-PCR** | pos | - | - | neg | neg |
|  | **DRMs (%)** | wt | - | - | ns | ns |
| **25** | **VL (copies/ml)** | 2.2x10^3^ | - | - | - | - |
|  | **RT-PCR** | pos | - | - | neg | neg |
|  | **DRMs (%)** | wt | - | - | ns | ns |
| **26** | **VL (copies/ml)** | 4.5x10^1^ | - | - | - | - |
|  | **RT-PCR** | pos | - | - | neg | neg |
|  | **DRMs (%)** | wt | - | - | ns | ns |
| **27** | **VL (copies/ml)** | 8.5x10^3^ | - | - | - | - |
|  | **RT-PCR** | pos | - | - | neg | neg |
|  | **DRMs (%)** | wt | - | - | ns | ns |
| **28** | **VL (copies/ml)** | 1.1x10^5^ | - | - | - | 5,6x10^4^ |
|  | **RT-PCR** | pos | - | - | neg | pos |
|  | **DRMs (%)** | wt | - | - | ns | wt |
| **29** | **VL (copies/ml)** | 1.8x10^4^ | - | - | - | 1,5x10^3^ |
|  | **RT-PCR** | pos | - | - | neg | pos |
|  | **DRMs (%)** | wt | - | - | ns | wt |
| **30** | **VL (copies/ml)** | 9.7x10^3^ | - | - | - | - |
|  | **RT-PCR** | pos | - | - | neg | - |
|  | **DRMs (%)** | wt | - | - | ns | - |
| **31** | **VL (copies/ml)** | 2.6x10^3^ | - | - | - | - |
|  | **RT-PCR** | pos | - | - | neg | - |
|  | **DRMs (%)** | wt | - | - | ns | - |
| **32** | **VL (copies/ml)** | 9.2x10^3^ | - | - | - | - |
|  | **RT-PCR** | pos | - | - | neg | - |
|  | **DRMs (%)** | wt | - | - | ns | - |
| **33** | **VL (copies/ml)** | 2.0x10^3^ | - | - | nd | - |
|  | **RT-PCR** | pos | - | - | pos | - |
|  | **DRMs (%)** | wt | - | - | wt | - |
| **34** | **VL (copies/ml)** | 1.7x10^4^ | - | - | - | - |
|  | **RT-PCR** | pos | - | - | neg | - |
|  | **DRMs (%)** | wt | - | - | ns | - |
| **35** | **VL (copies/ml)** | 5.9x10^5^ | - | - | - | - |
|  | **RT-PCR** | pos | - | - | - | neg |
|  | **DRMs (%)** | wt | - | - | - | ns |
| **36** | **VL (copies/ml)** | 1.9x10^3^ | - | - | - | - |
|  | **RT-PCR** | pos | - | - | - | neg |
|  | **DRMs (%)** | wt | - | - | - | ns |
| **37** | **VL (copies/ml)** | 3.4x10^2^ | - | - | - | - |
|  | **RT-PCR** | neg | - | - | neg | neg |
|  | **DRMs (%)** | ns | - | - | ns | ns |
| **38** | **VL (copies/ml)** | 4.0x10^2^ | - | - | - | - |
|  | **RT-PCR** | neg | - | - | neg | neg |
|  | **DRMs (%)** | ns | - | - | ns | ns |
| **39** | **VL (copies/ml)** | 5.4x10^2^ | - | - | - | - |
|  | **RT-PCR** | neg | - | - | neg | neg |
|  | **DRMs (%)** | ns | - | - | ns | ns |
| **40** | **VL (copies/ml)** | 2.5x10^2^ | - | - | - | - |
|  | **RT-PCR** | neg | - | - | neg | neg |
|  | **DRMs (%)** | ns | - | - | ns | ns |
| **41** | **VL (copies/ml)** | 1.7x10^2^ | - | - | - | - |
|  | **RT-PCR** | neg | - | - | neg | neg |
|  | **DRMs (%)** | ns | - | - | ns | ns |
| **42** | **VL (copies/ml)** | nd | - | - | - | - |
|  | **RT-PCR** | neg | - | - | neg | neg |
|  | **DRMs (%)** | ns | - | - | ns | ns |
| **43** | **VL (copies/ml)** | nd | - | - | - | - |
|  | **RT-PCR** | neg | - | - | neg | neg |
|  | **DRMs (%)** | ns | - | - | ns | ns |
| **44** | **VL (copies/ml)** | 1.9x10^2^ | - | - | - | - |
|  | **RT-PCR** | neg | - | - | neg | neg |
|  | **DRMs (%)** | ns | - | - | ns | ns |
| **45** | **VL (copies/ml)** | nd | - | - | - | - |
|  | **RT-PCR** | neg | - | - | neg | neg |
|  | **DRMs (%)** | ns | - | - | ns | ns |
| **46** | **VL (copies/ml)** | nd | - | - | - | - |
|  | **RT-PCR** | neg | - | - | neg | neg |
|  | **DRMs (%)** | ns | - | - | ns | ns |
| **47** | **VL (copies/ml)** | nd | - | - | - | - |
|  | **RT-PCR** | neg | - | - | neg | neg |
|  | **DRMs (%)** | ns | - | - | ns | ns |
| **48** | **VL (copies/ml)** | nd | - | - | - | - |
|  | **RT-PCR** | neg | - | - | neg | - |
|  | **DRMs (%)** | ns | - | - | ns | - |
| **49** | **VL (copies/ml)** | nd | - | - | - | - |
|  | **RT-PCR** | neg | - | - | - | neg |
|  | **DRMs (%)** | ns | - | - | - | ns |
